# Liver iron concentration thresholds: Where do they really come from?

**DOI:** 10.1002/hem3.70122

**Published:** 2025-04-04

**Authors:** Lukas Müller, Diego Hernando, Moniba Nazeef, Scott B. Reeder

**Affiliations:** ^1^ Department of Radiology University of Wisconsin–Madison Madison Wisconsin USA; ^2^ Department of Radiology University Medical Center Mainz Mainz Germany; ^3^ Department of Medical Physics University of Wisconsin–Madison Madison Wisconsin USA; ^4^ Department of Biomedical Engineering University of Wisconsin–Madison Madison Wisconsin USA; ^5^ Department of Electrical and Computer Engineering University of Wisconsin–Madison Madison Wisconsin USA; ^6^ Department of Medicine, Division of Hematology/Oncology University of Wisconsin School of Medicine and Public Health Madison Wisconsin USA; ^7^ UW Carbone Cancer Center, University of Wisconsin School of Medicine and Public Health Madison Wisconsin USA; ^8^ Department of Medicine University of Wisconsin–Madison Madison Wisconsin USA; ^9^ Department of Emergency Medicine University of Wisconsin–Madison Madison Wisconsin USA

Systemic iron overload arises from a variety of causes, including genetic disorders of iron absorption, repeated blood transfusions, hemolytic anemias, hematologic malignancies, and chronic liver disease, among others.^1^ The body lacks mechanisms for active elimination of excess iron, leading to accumulation in the liver, spleen, pancreas, endocrine glands, bone marrow, and heart. Excess iron is toxic and leads to organ dysfunction and early mortality, typically from heart failure or end‐stage liver disease.^2^

Treatment for iron overload aims to prevent complications through therapeutic phlebotomy or chelation, depending on the underlying etiology.^3^ Early detection and quantification of total body iron (TBI) stores are critical for timely intervention before irreversible damage occurs. Importantly, phlebotomy and chelation have notable side effects and high costs.^4^ For these reasons, accurate monitoring of TBI is essential to initiate and monitor treatment. Although serum ferritin (SF) is the simplest means to assess TBI, it is an acute phase reactant often confounded by unrelated factors and may not accurately reflect TBI. Moreover, up to 30% of patients exhibit a discrepancy in their response to chelation therapy as assessed by changes in SF and liver iron concentration (LIC).^5^

Importantly, TBI is linearly and highly correlated with LIC. LIC is widely accepted as a surrogate of TBI,^6^ and its accurate measurement leads to informed objective management strategies.^7^ For this reason, LIC measurement is included in current guidelines for the surveillance and treatment of systemic iron overload.^1^, ^2^, ^8^

Historically, LIC has been assessed using non‐targeted biopsy combined with spectrophotometric assays.^9^ LIC can be reported interchangeably as milligrams of iron per gram of dry liver tissue (mg Fe/g dry, or mg/g) or micromoles of iron per gram of dry tissue (μmol Fe/g dry, or μmol/g).^2^ Although biopsy is accepted as the reference to assess LIC, it is invasive and expensive, suffers from sampling variability, and is contraindicated in patients with bleeding diatheses.^10^ Fortunately, LIC can be assessed noninvasively with high accuracy and precision using state‐of‐the‐art magnetic resonance imaging (MRI).^2^, ^6^

St Pierre et al.^11^ summarized LIC thresholds as follows: <1.8 mg/g, normal; 3.2 mg/g, the lower limit of the optimal range for chelation therapy; 7.0 mg/g, the upper limit of the optimal range for chelation therapy; >7.0 mg/g, increased risk of complications including liver fibrosis and diabetes; >15.0 mg/g, greatly increased risk for cardiac disease and early death. Current patient management guidelines rely on these thresholds^1^, ^8^ and are widely used for clinical decision‐making. But do we know where they come from and the evidence behind their use?

The LIC threshold of 1.8 mg/g, often cited as upper threshold of “normal,” is based on a single study from 1986^12^ established using "autopsy liver biopsy specimens obtained from 40 subjects who had suffered sudden death,” with no other detail.^12^ Another frequently cited study defined the “normal” LIC range as 0.6–1.2 mg/g^4^, ^13^ using a Superconducting Quantum Interference Device (SQUID) in “twenty normal hospital personnel.”^14^ Importantly, these authors did not propose any thresholds. Rather, we assume this range was inferred from figure 2 of this work,^14^ by Olivieri and Brittenham in a review article published in 1997,^13^ which serves as the basis for subsequent guidelines.

LIC values in the range of 3.2–7.0 mg/g are considered “optimal” for chelation therapy,^4^, ^13^ intended to “minimize both the risk of adverse effects from the iron‐chelating agent and the risk of complications from iron overload.”^13^ Remarkably, this widely accepted “optimal” window for chelation therapy in patients with transfusion‐dependent *thalassemia* was derived solely in patients with *heterozygous hemochromatosis*.^1^, ^15^, ^16^

Specifically, the lower threshold of 3.2 mg/g is based on a single study by Cartwright et al.^15^ in 145 patients with heterozygous hemochromatosis. No patient received chelation therapy, none had ill effects of iron overload, and all had normal life expectancy. Importantly, this group had a mean LIC of 3.2 mg/g, as cited by two influential review articles,^4^, ^13^ establishing 3.2 mg/g as the lower threshold for chelation treatment for *all etiologies* of iron overload. It is noteworthy that the 1.8–3.2 mg/g range remains entirely undefined. Although the evidence from Cartwright et al. is relatively weak, the rationale for withholding chelation therapy in patients with LIC less than 3.2 mg/g has merit.

The upper threshold of 7.0 mg/g defining the range for “optimal” chelation therapy is based on studies that do not specify any threshold values.^17^, ^18^, ^19^ Rather, in their review articles, Olivieri et al. and Kushner et al.^4^, ^13^ extrapolated the original data^17^, ^18^, ^19^ to establish an association of this threshold with complications. Specifically, they asserted that patients with LIC exceeding 7.0 mg/g had an increased risk of diabetes and liver fibrosis.^4^, ^13^ However, based on our review, we could only identify data that demonstrate an increased risk of liver fibrosis when LIC exceeds 7.0 mg/g (figure 1 in Loréal et al.^19^). None of the original studies^17^, ^18^, ^19^ demonstrated an association between LIC and risk of diabetes. Notably, a recent cohort study^20^ on transfusion‐dependent thalassemia on 427 patients found that an LIC > 8.0 mg/g was associated with an increased risk of cardiovascular‐related and all‐cause mortality.

When LIC exceeds 15.0 mg/g, patients are at “greatly increased risk for iron‐induced cardiac disease and early death,” and treatment escalation is recommended.^13^ This threshold ultimately traces back to a single paper from 1994.^21^ In this study, Brittenham et al. investigated the risk of premature death and cardiac complications in 59 patients with *thalassemia major*, but do not mention a threshold of 15.0 mg/g. We assume this threshold is inferred by Olivieri et al. and Kushner et al.^4^, ^13^ from the data reported by Brittenham et al.^21^ Specifically, figure 2 from Brittenham et al.^21^ indicates that a ratio of transfusional iron load to deferoxamine use exceeding 0.6 increases the risk of cardiac complications and premature death. Extrapolating from figure 1, a ratio exceeding 0.6 corresponds to LIC of 15.0 mg/g or higher.^21^ We presume this to be the origin of this widely cited threshold.

Table 1 provides a nested summary of current guidelines, review articles on which guidelines are based, and foundational references that provide original sources of data used by the review articles and guidelines to establish the LIC thresholds discussed earlier. Notably, other reviews and expert recommendations suggest alternative cutoffs, without direct reference to supporting data.^22^, ^23^

**Table 1:** Summary of relevant literature, including recent guidelines, review articles, and foundational references from which commonly used LIC thresholds were derived. Evidence from which these thresholds are derived is also summarized.

	Main topic/note(s)	Referenced work
**Guidelines**		
Thalassaemia International Foundation (2025) (doi: n/a, see link below^a^)	General guidelines for the management of transfusion‐dependent β‐thalassemia	Olivieri and Brittenham (1997)
British Society for Haematology (2021) (doi: 10.1111/bjh.17839)	General guidelines for monitoring and management of iron overload for hemoglobinopathies and rare anemias	Kushner et al. (2001)
Review and Guidelines from the ESGAR and SAR (2023) (doi: 10.1148/radiol.221856)	Review of LIC quantification with MRI, guidelines for recommended use of MRI	St Pierre et al. (2006)
**Review articles on LIC cited by guidelines** b		
St Pierre et al. (2006) (doi: 10.1182/blood‐2004‐01‐0177)	Review of LIC quantification with MRI	Bassett et al. (1986) Olivieri and Brittenham (1997)
Olivieri and Brittenham (1997) (doi: 10.1182/blood.V89.3.739)	Review of iron‐chelating therapy and treatment of thalassemia	Brittenham et al. (1982) Cartwright et al. (1979) Niederau et al. (1985) Niederau et al. (1996) Loréal et al. (1992) Brittenham et al. (1994)
Kushner et al. (2001) (doi: 10.1182/asheducation‐2001.1.47)	Review of management of secondary iron overload	Brittenham et al. (1982) Cartwright et al. (1979) Niederau et al. (1985) Niederau et al. (1996) Loréal et al. (1992) Brittenham et al. (1994)
**Foundational references used by review articles and guidelines**		**Threshold**
Bassett et al. (1986) (doi: 10.1002/hep.1840060106)	LIC quantification in early hemochromatosis, evidence of “normal” LIC based on “40 subjects who had suffered sudden death”	<1.8 mg/g
Brittenham et al. (1982) (doi: 10.1056/NEJM198212303072703)	Measurements of human iron stores, “normal” SQUID‐based LIC based on “twenty normal hospital personnel”	
Cartwright et al. (1979) (doi: 10.1056/NEJM197907263010402)	Phenotypic expression of hemochromatosis, group of heterozygotes, none receiving chelation therapy, with a mean LIC of 3.2 mg/g and no apparent ill effects, normal life expectancy	3.2 mg/g
Niederau et al. (1985) (doi: 10.1056/NEJM198511143132004)	Survival and death of noncirrhotic patients with hemochromatosis, no LIC mentioned	7.0 mg/g
Niederau et al. (1996) (doi: 10.1053/gast.1996.v110.pm8613000)	Long‐term survival in patients with hereditary hemochromatosis; table 7 shows that patients with stage 1 fibrosis (*n* = 10) had a LIC of 13.9 mg/g (while patients without fibrosis (*n* = 7) had a LIC of 11.6 mg/g in their study), no threshold for LIC identified	
Loréal et al. (1992) (doi: 10.1016/s0168‐8278(05)80104‐7)	Liver fibrosis in hemochromatosis correlates with LIC; threshold around 7.0 mg/g can be derived from figure 1	
Brittenham et al. (1994) (doi: 10.1056/NEJM199409013310902)	Efficacy of deferoxamine in patients with thalassemia major, no threshold defined in original article, 15.0 mg/g threshold later cited in review articles likely derived from transfusional iron load:deferoxamine (TILD) ratio and correlation to early death and cardiac complications (figure 2), with correlation of the TILD ratio to LIC > 15.0 mg/g (figure 1)	15.0 mg/g

Abbreviations: ESGAR, European Society of Gastrointestinal and Abdominal Radiology; LIC, liver iron concentration; MRI, magnetic resonance imaging; SAR, Society of Abdominal Radiology.

^a^ https://thalassaemia.org.cy/publications/tif-publications/guidelines-for-the-management-of-transfusion-dependent-β-thalassaemia-5th-edition-2025/.

^b^ These articles are either directly or indirectly cited by the guideline articles.

In our view, the level of evidence^24^ that supports current LIC thresholds is low. These thresholds are based on sparse or indirect data derived from small patient populations at single centers in narrow patient populations, raising questions about the applicability and generalizability of these thresholds. This gap in knowledge represents a significant need to reevaluate the validity of these thresholds and their clinical utility, based on larger and more diverse datasets.

Although liver biopsy is no longer widely used, quantitative MRI methods are increasingly available as commercial applications from all major MRI vendors.^2^ Modern MRI methods can assess LIC accurately and precisely within a 20 s breath‐hold, at a cost similar to ultrasound.^25^ It is noteworthy that MRI‐based LIC estimates are highly correlated and fully calibrated with biopsy‐based LIC measurements on all major vendors and at commonly used field strengths. Thus, MRI‐based LIC estimates and thresholds are equivalent to those determined by biopsy. In this way, modern quantitative MRI methods provide a viable and standardized means to collect comprehensive data across a wide spectrum of iron overload etiologies in diverse patient populations that suffer from iron overload. The increasingly widespread availability of MRI‐based LIC measurements presents a valuable opportunity for the hematology and radiology communities to collaborate to develop a broader strategy. Such a strategy might include registries or other mechanisms needed to refine and reassess the generalizability and applicability of LIC thresholds that are used in treatment decisions and their impact on prognosis. We invite experts in both fields to join in this effort.

## AUTHOR CONTRIBUTIONS

All authors drafted and revised the manuscript content.

## CONFLICT OF INTEREST STATEMENT

The authors wish to acknowledge support from GE Healthcare who provide research support to the University of Wisconsin. Furthermore, Dr. Reeder is supported by the John H. Juhl Endowed Chair of Radiology. Moniba Nazeef is supported by the Kathy Mosher faculty fellowship in Sickle Cell Disease.

## FUNDING

This research received no funding.

## Data Availability

No new data were created or analyzed in this study. The research is based entirely on previously published literature, all of which is publicly available and cited accordingly.
